# Taxonomy and systematics of 2 new species of myxozoans (Cnidaria: Myxobolidae) parasitizing the gills of *Iheringichthys labrosus* (Teleostei: Pimelodidae) from southeastern Brazil

**DOI:** 10.1017/S0031182025100589

**Published:** 2025-09

**Authors:** Diego Henrique Mirandola Dias Vieira, Maria João Santos, Sónia Rocha, Luis Filipe Rangel, Rodrigo Bravin Narciso, Reinaldo José da Silva

**Affiliations:** 1 Division of Parasitology, São Paulo State University (UNESP), Institute of Biosciences, Botucatu, SP, Brazil; 2 Department of Biology, Faculty of Sciences, CIIMAR, University of Porto, Porto, Portugal; 3 CIIMAR/CIMAR LA, Interdisciplinary Centre of Marine and Environmental Research, Laboratory of Animal Parasitology and Pathology, University of Porto, Matosinhos, Portugal; 4 Instituto de Investigação e Inovação em Saúde (i3S), University of Porto, Porto, Portugal; 5 School of Medicine and Biomedical Sciences (ICBAS), University of Porto, Porto, Portugal

**Keywords:** gill infection, myxozoa, Pardo River, phylogeny, ultrastructure

## Abstract

During a survey of myxozoan infections in fishes from the Pardo River, Paranapanema River basin, São Paulo State, Brazil, 2 new species – *Henneguya avareensis* n. sp. and *Myxobolus iheringichthys* n. sp. – were discovered parasitizing the gills of *Iheringichthys labrosus*, a commercially important pimelodid fish in South America. Species descriptions were based on the morphology of myxospores and partial sequences of the small subunit ribosomal DNA. Phylogenetic analysis revealed host-related clustering, with the new species clustering together with other myxobolids that parasitize Pimelodidae (Siluriformes). *Myxobolus iheringichthys* n. sp. clustered specifically with *Myxobolus cordeiroi*, together forming yet another lineage of myxobolids infecting Pimelodidae fishes. Our analysis underscores the importance of monitoring the presence of these parasites in stocks of *I. labrosus* to assess potential pathologies they may cause. This is the first report of myxozoans parasitizing the gills of this Neotropical catfish.

## Graphical abstract

**Figure:**
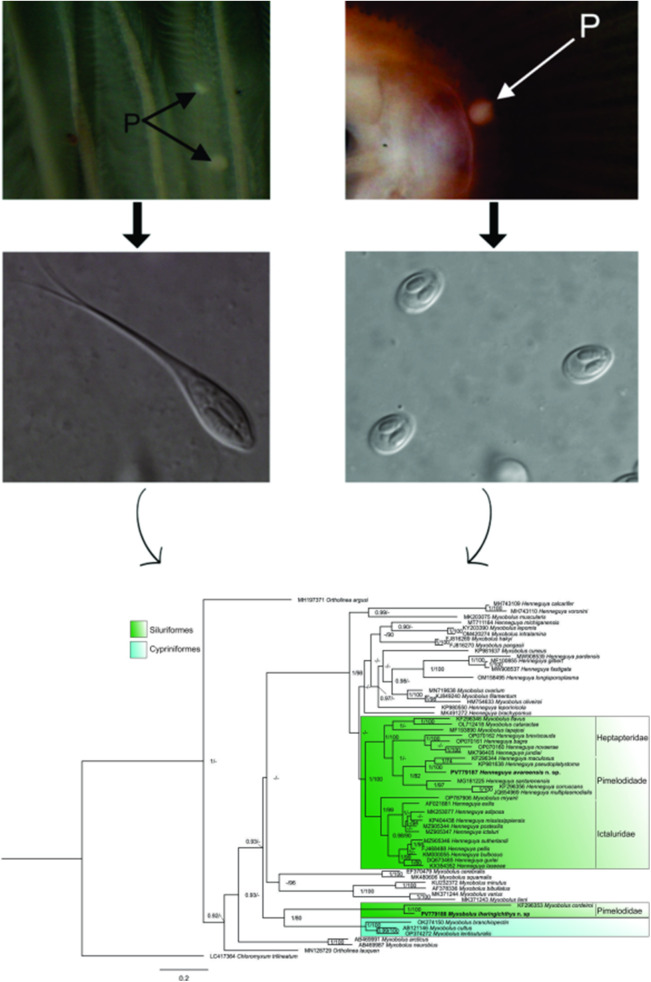

## Introduction

Myxozoans are highly diverse microscopic parasites that belong to the phylum Cnidaria. They are ubiquitous in aquatic environments, and can infect a wide variety of hosts, including vertebrates (usually fishes) and invertebrates (annelids and bryozoans) (Rangel et al., 2015). During their complex life cycles, these parasites develop morphologically different stages that range from waterborne-infective spores to highly specialized forms living in host tissues or organ cavities (Adlard et al., 2015). The taxonomy of myxozoans is a challenging discipline due to the morphological complexity and diversity of these microscopic parasites. The traditional classification of myxozoans is primarily based on the morphological characteristics of spores, such as presence/absence of valvular processes and organization of polar capsules. However, advances in molecular biology have allowed for a more integrative approach to myxozoan taxonomy, revealing insights into their phylogeny and genetic diversity (Fiala, 2006).

*Myxobolus* Bütschli, 1882 and *Henneguya* Thélohan, 1892 are myxozoan genera belonging to the subclass Myxosporea Bütschli, 1881 (Lom and Dyková, 2006). Currently, approximately 980 *Myxobolus* spp. and 260 *Henneguya* spp. are known (Eiras et al., 2021; Rangel et al., 2023). Both genera are known to cause diseases in fish, negatively impacting aquaculture industry and the health of aquatic ecosystems (Banu and Rathinam, 2023). Studies have investigated the genetic diversity, ecology and transmission strategies of these myxosporeans, aiming to develop more effective control and prevention methods (Palenzuela et al., 2002). The information obtained is crucial for the sustainable management of aquatic resources and for mitigating the impacts of diseases caused by these parasites. Phylogenetic studies support the paraphyletic nature of the *Myxobolus* clade, which contains not only *Myxobolus* spp. but also numerous *Henneguya* spp., as well as species of the genera *Thelohanellus, Cardimyxobolus, Hennegoides, Dicauda, Unicauda* and *Triangula* (Liu et al., 2010, 2019).

*Iheringichthys labrosus* (Lütken, 1874) is a freshwater fish species belonging to the family Pimelodidae (order Siluriformes), commonly known as ‘mandi-beiçudo’ or ‘mandi-bicudo’. It is found in rivers of South America, such as the Paraná and Paraguay River basins (Lundberg and Littmann, 2003). It is an important fish for both sport and commercial fishing, being used for human consumption in some riverside communities (Abes et al., 2001; Kohn and Fernandes, 2011). Few parasitological studies of *I. labrosus* have been performed (França et al., 2003; Moreira et al., 2005; Kohn and Fernandes, 2011), none of which are related to myxozoan parasites.

During a survey targeting the biodiversity of myxozoans infecting fishes from the Pardo River in São Paulo State (Brazil), 2 new species of these cnidarian parasites were found parasitizing the gills of *I. labrosus*. In this study, a thorough taxonomic description of the new species is performed including morphological, molecular and phylogenetic data, thus contributing to a better understanding of the biodiversity of myxozoans in South America. It is noteworthy that these 2 species constitute the first records of myxozoans parasitizing this Neotropical catfish.

## Materials and methods

### Host collection

Specimens of *I. labrosus* (*n* = 10, sex undetermined, ranging from 14.1 to 22.4 cm in length, and 210 to 289 g in weight) were captured from the Pardo River (22°57ʹ48.35″ S; 48°47ʹ26.07″ W), in the municipality of Avaré, São Paulo State, Brazil, between 2021 and 2022. Authorization for capture was provided by the Instituto Chico Mendes de Conservação da Biodiversidade (SISBIO #60640-1). Fish were captured using casting nets, euthanized with sodium thiopental (Thiopentax®), measured and weighed, and dissected for the macro- and microscopic examination of internal organs. All procedures adhered to the guidelines set forth by the Ethical Commission for Animal Experimentation at São Paulo State University (UNESP), Institute of Biosciences, Botucatu, Brazil (CEUA/IBB/UNESP n^o^ 9415260520).

### Myxozoan collection and morphological analysis

Macro- and microscopic examination of fresh host organs focused on the gills, stomach, intestine, heart, liver, kidney, swim bladder and gallbladder, to detect the presence of myxozoans. This assessment was conducted utilizing a Leica S6 D stereomicroscope (Leica Microsystems, Germany) with a 16× ocular lens. Gills infected by myxozoan plasmodia were selectively sampled for both morphological and molecular analyses. Prevalence of infection was determined following the methodology outlined by Bush et al. (1997).

Fresh gill smears containing myxozoan plasmodia were individually examined under a light microscope (Leica DM1000) for performing morphological analyses. Measurements of fresh myxospores were determined following the guidelines established by Lom and Arthur (1989). Thirty myxospores from each myxozoan species were measured using a Leica DMLB 5000 microscope equipped with differential interference contrast optics (Leica Microsystems, Wetzlar, Germany), at a magnification of 1000×. In both cases, morphometric data were collected from the myxospores of a single plasmodium, which followed for molecular analysis. Myxospore measurements are expressed in micrometres, and include average, standard deviation (s.d.) and range within parentheses. Digital images were captured using the Leica LAS V3.8 software applications (Leica Microsystems, Germany).

The procedures used for transmission electron microscopy mainly followed the protocol described by Casal et al. (2023). Briefly, small portions of gills containing plasmodia were fixed in 3% glutaraldehyde buffered in 0.2 M sodium cacodylate (pH 7.4) for 20–24 h, and post-fixed in 2% osmium tetroxide in the same buffer for 3–4 h, while being kept at 4 °C. Samples were then dehydrated in a graded series of ethanol, and embedded using ascending mixtures of epoxy resin in oxide propylene, ending in epoxy resin. Semithin sections were stained with methylene blue-Azure II. Ultrathin sections were double contrasted using uranyl acetate and lead citrate, prior to being observed and photographed using a JEOL 100 CXII TEM (JEOL Optical, Tokyo, Japan), operated at 60 kV.

### Molecular analysis

Isolated plasmodia were carefully removed from the gills and immediately fixed in absolute ethanol. Authorization for genetic data access was obtained from the Brazilian Ministry of Environment (Sisgen AAE660A). DNA extraction followed the DNeasy® Blood and Tissue kit (animal tissue protocol) (QIAGEN Inc., California, USA) instructions, and the final DNA concentration was quantified using a NanoDrop 2000 spectrophotometer (Thermo Fisher Scientific, Massachusetts, USA) at 260 nm.

For amplifying partial sequences of the small subunit ribosomal DNA (ssrDNA), the primer pairs ERIB1 (5′‐ACCTGGTTGATCCTGCCAG‐3′) (Barta et al., 1997)–ACT1r (5′‐AATTTCACCTCTCGCTGCCA‐3′) (Kent et al., 2000), and Myxgen4F (5′‐GTGCCTTGAATAAATCAGAG‐3′) (Hallett and Diamant, 2001)–ERIB10 (5′‐CTTCCGCAGGTTCACCTACGG‐3′) (Barta et al., 1997) were used. Polymerase chain reactions (PCRs) were performed with a final volume of 25 μL, by adding 20–40 ng of DNA, 1 μL of each primer at 10 pmol and 20 µL of ultrapure water to PCR Ready-to-Go beads (Pure TaqTMReady-to-GoTM beads, GE Healthcare, Chicago, USA). When a bead is reconstituted to a final volume of 25 μL, the concentration of each dNTP is 200 μM in 10 mM Tris-HCl (pH 9.0 at room temperature), 50 mM KCl and 1.5 mM MgCl_2_. Amplifications were conducted on a Bio-Rad MJ Mini Gradient Thermal Cycler (Bio-Rad Laboratories, PA, USA), with cycling parameters comprising an initial denaturation at 95 °C for 3 min, followed by 35 cycles of denaturation at 95 °C for 1 min, annealing at 55 °C for 45 s, extension at 72 °C for 2 min and a final extension at 72 °C for 7 min. PCR products were visualized on a 1% agarose gel stained with GelRed, and compared with a 1 kb Plus DNA Ladder (Invitrogen, Thermo Fisher Scientific, Massachusetts, USA). Positive PCR products were purified with magnetic beads from the Ampure XP kit (Beckman Coulter) following the manufacturer’s protocol and sequenced using the same set of PCR amplification primers. Sequencing reactions were conducted using the BigDye® Terminator v3.1 Cycle Sequencing Kit (Applied Biosystems) and analysed by capillary electrophoresis on the ABI3730xl Genetic Analyzer (Applied Biosystems, Foster City, CA, USA).

The ssrDNA partial sequences obtained for each myxozoan species analysed were assembled using Sequencher v. 5.2.4 (Gene Codes, Ann Arbor, MI, USA), prior to being aligned with their most similar sequences available in the GenBank database, according to the Basic Local Alignment Search Tool (BLAST). The dataset used for performing the phylogenetic analysis included the ssrDNA sequences of species exceeding 80% of genetic similarity to the myxozoans in study. The ssrDNA sequence of *Chloromyxum trilineatum* (LC417364) and *Ortholinea lauquen* (MN128729) served as outgroup. Sequences were aligned using the ClustalW algorithm (Larkin et al., 2007) with the default settings selected in Geneious 7.1.3 software. The similarity between was compared by similarity matrix in Geneious 7.1.3 software. The Bayesian inference method was performed in MrBayes 3.1.2 (Ronquist and Huelsenbeck, 2003), with the Markov Chain Monte Carlo tree searches conducted in parallel runs for 5 million generations each. The ‘burn-in’ was set at 25%. PhyML 3.1 (Guindon et al., 2010) software was used to perform Maximum Likelihood analysis, with bootstrap confidence calculated with 1000 replications and the GTR + I + G evolutionary model, which was chosen by jModeltest (Posada, 2008) as the best model for the analysis. The resulting trees were visualized using Figtree 1.4.2. (Rambaut, 2012).

## Results

Two species belonging to the Myxobolidae family were observed in the gill arch and filaments of *I. labrosus*, having been morphologically differentiated as belonging to the genera *Myxobolus* and *Henneguya*. Comparisons of biological, morphological and molecular data support their description as new species. There was no coinfection. Only one plasmodium containing myxospores of the genus *Myxobolus* was found, which is the reason why no other morphological analyses of this species were carried out.

**Description of *Myxobolus iheringichthys* n. sp.**

Phylum Cnidaria Hatschek, 1888

Subphylum Endocnidozoa Zrzavý and Hypša, 2003

Class Myxozoa Grassé, 1970

Subclass Myxosporea Bütschli, 1881

Order Bivalvulida Shulman, 1959

Family Myxobolidae Thélohan, 1892

Genus *Myxobolus* Bütschli, 1882

*Myxobolus iheringichthys* n. sp.

**Figure 1.**
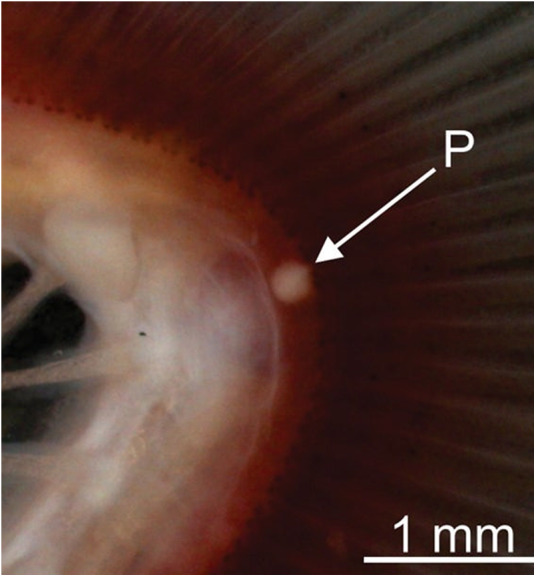
Plasmodium (P) of *Myxobolus iheringichthys* n. sp. found parasitizing the gill arch of *Iheringichthys labrosus* collected from the Pardo River, municipality of Avaré, State of São Paulo, Brazil.

**Figure 2.**
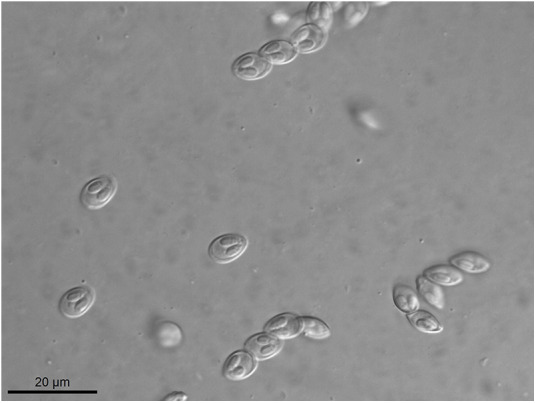
Light micrograph of fresh myxospores of *Myxobolus iheringichthys* n. sp. found parasitizing the gill arch of *Iheringichthys labrosus* collected from the Pardo River, municipality of Avaré, State of São Paulo, Brazil.

**Figure 3.**
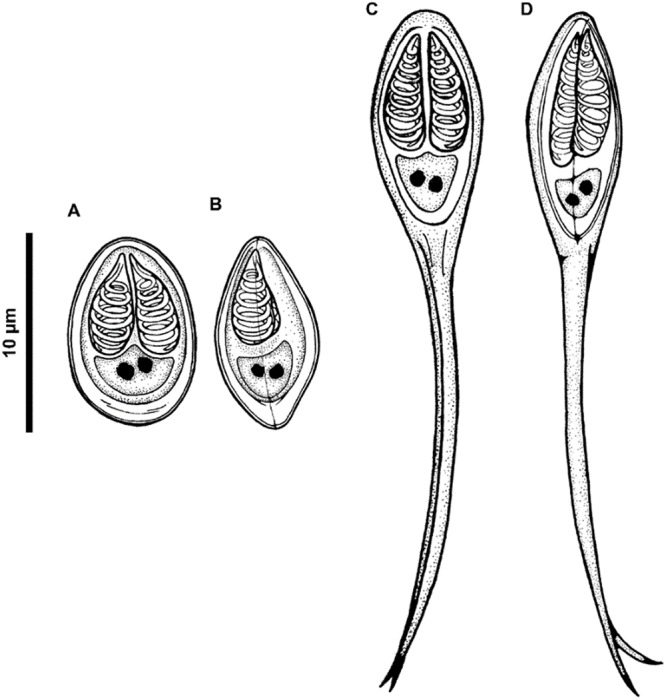
**A-B.** Schematic drawing of the myxospores of *Myxobolus iheringichthys* n. sp. In frontal (A) and side (B) view. (C and D). Schematic drawing of the myxospores of *Henneguya avareensis* n. sp. In frontal (C) and side (D) view.

A single plasmodium was observed in the gill arch (Figure 1), whitish, round and measuring about 0.1 mm. Myxospores oval, formed by 2 smooth and symmetric valves united along a prominent suture line. Myxospores measuring 8.9 ± 0.3 (8.2–9.5) μm in length, 5.8 ± 0.3 (5.5–6.4) μm in width and 4.4 ± 0.2 (4.1–4.8) μm in thickness. Two polar capsules located at the anterior end, slightly convergent at the apex, equal in size, pyriform and measuring 4.3 ± 0.3 (3.7–4.6) μm in length and 2.0 ± 0.2 (1.6–2.4) μm in width. Polar tubules with 6–7 turns. Binucleate sporoplasm (Figures 2; 3A).

### Taxonomic summary

Type-host: *Iheringichthys labrosus* (Lütken, 1874) (Siluriformes, Pimelodidae).

Type-locality: Pardo River (22°57ʹ48.35″ S 48°47ʹ26.07″ W), Paranapanema River basin, municipality of Avaré, São Paulo State, Brazil.

Site of infection: histozoic, gill arch.

Prevalence of infection: 10% (1 infected out of 10 fish examined).

Type-material: A glass slide with myxospores (hapantotype) was deposited in the collection of the Instituto Nacional de Pesquisa da Amazônia (INPA), Brazil (No. INPA-CND 000113). The ssrDNA partial sequence was deposited in GenBank with accession number PV779188.

Etymology: The specific epithet name is derived from the host genus.

Zoobank ID: urn:lsid:zoobank.org:pub:812FDDC2-9BBB-4 FFB-8C19-E778CC7ABF16

**Description of *Henneguya avareensis* n. sp.**

Phylum Cnidaria Hatschek, 1888

Subphylum Endocnidozoa Zrzavý and Hypša, 2003

Class Myxozoa Grassé, 1970

Subclass Myxosporea Bütschli, 1881

Order Bivalvulida Shulman, 1959

Family Myxobolidae Thélohan, 1892

Genus *Henneguya* Thélohan, 1892

*Henneguya avareensis* n. sp.

**Figure 4.**
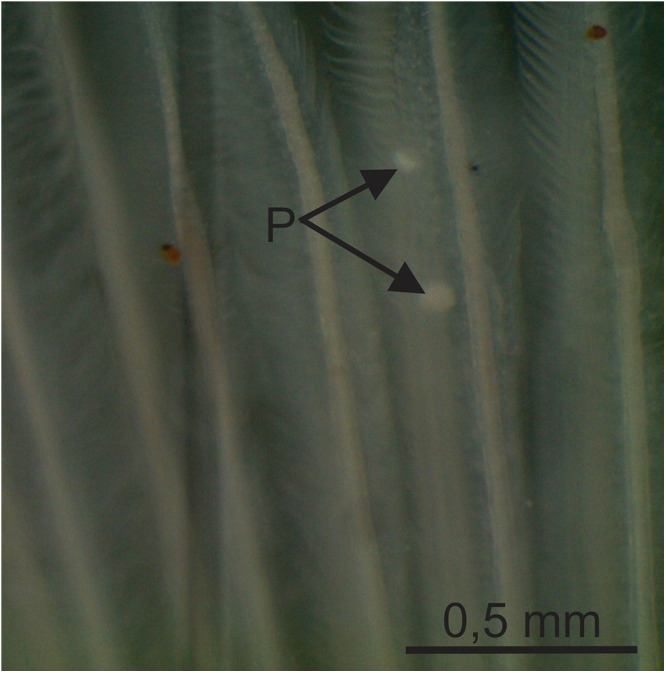
Plasmodia (P) of *Henneguya avareensis* n. sp. found parasitizing the gill filaments of *Iheringichthys labrosus* collected in the Pardo River, municipality of Avaré, State of São Paulo, Brazil.

**Figure 5.**
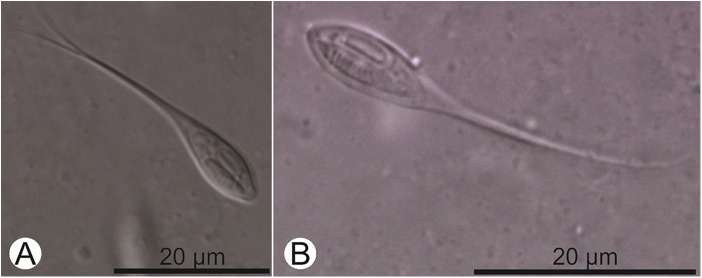
(A and B) light micrographs of fresh myxospores of *Henneguya avareensis* n. sp. found parasitizing the gill filaments of *Iheringichthys labrosus* collected in the Pardo River, municipality of Avaré, State of São Paulo, Brazil.

Plasmodia located in the gill filaments, whitish, rounded and measuring about 0.1 mm (Figure 4). Myxospore body elongated and convex-shaped, formed by 2 smooth and symmetric valves, each with a caudal appendage extending posteriorly. Myxospores measuring 34.8 ± 1.5 (32.3–36.3) μm in total length, 11.9 ± 0.4 (11.2–12.4) μm in body length, 5.4 ± 0.5 (4.7–6.0) μm in body width and 4.1 ± 0.1 (3.9–4.2) μm in body thickness. Caudal appendages measuring 23.0 ± 1.7 (20.0–25.0) μm in length. Two polar capsules located at the anterior end, equal in size, pyriform and measuring 6.4 ± 0.3 (6.0–6.6) μm in length and 2.1 ± 0.1 (2.0–2.2) μm in width. Polar tubules with 10–12 turns. Binucleate sporoplasm (Figures 3B; 5).

### Ultrastructure

**Figure 6.**
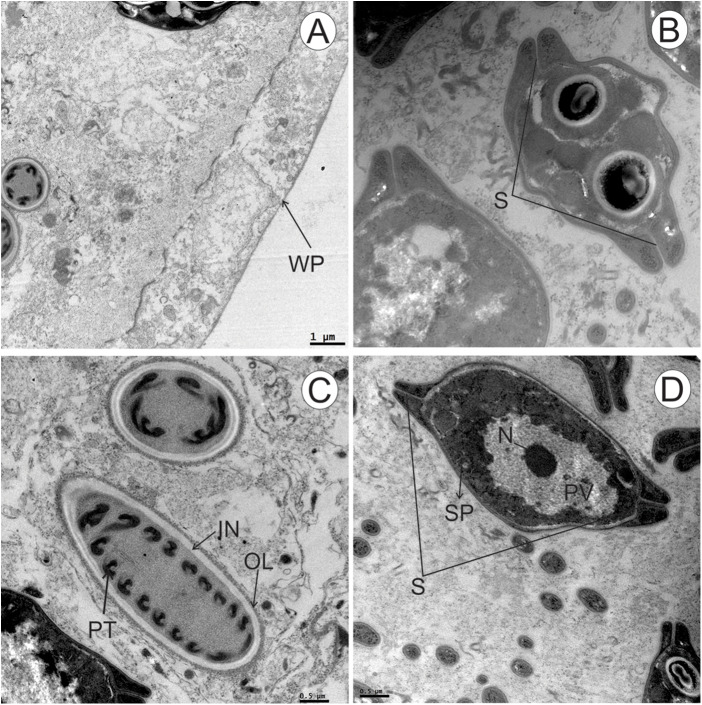
(A–D) TEM micrographs of the plasmodia and myxospores of *Henneguya avareensis* n. sp. Found in the gill filaments of *Iheringichthys labrosus*. (A) Plasmodial wall (WP) smooth. (B) Transverse section of a myxospore showing the sutures (S) with its thickened valves. (C) Longitudinal cut of a polar capsule. Notice the outer electron-dense layer (OL) and inner electron-lucent layer (IL) forming the wall, and the polar tubules (PT) coiled within. (D) Transverse section of a myxospore showing the posterior vacuole (PV) containing 1 of the nuclei (N). Note the heterogeneous content in the sporoplasm (SP) and sutures (S).

Plasmodia mostly comprising mature myxospores and bearing few cytoplasmic organelles. Plasmodial wall smooth (Figure 6A). Wall of the polar capsules formed by 2 layers – an inner electron-lucent layer surrounded by an outer electron-dense layer. Mature myxospores evidencing the 2 smooth valves united along a straight suture line (Figure 6B). Each polar capsule with an isofilar polar tubule located in its matrix, forming 10–12 coils around the inner wall (Figure 6C). Transverse sections of the caudal appendages showing the absence of discernible coating (Figure 6D). Sporoplasm displaying heterogeneous content surrounding a posterior vacuole and 2 nuclei (Figure 6B, D).

### Taxonomic summary

Type-host: *Iheringichthys labrosus* (Lütken, 1874) (Siluriformes, Pimelodidae).

Type-locality: Rio Pardo (22°57ʹ48.35″ S 48°47ʹ26.07″ W), Paranapanema River basin, municipality of Avaré, São Paulo State, Brazil.

Site of infection: histozoic, gill filaments, intrafilamental type.

Prevalence of infection: 60% (6 infected out of 10 fish examined).

Type-material: A glass slide with myxospores (hapantotype) was deposited in the collection of the Instituto Nacional de Pesquisa da Amazônia (INPA), Brazil (No. INPA-CND 000112). The ssrDNA partial sequence was deposited in GenBank with accession number PV779187.

Etymology: The specific epithet refers to the name of the city where the host was collected (Avaré).

Zoobank ID: urn:lsid:zoobank.org:pub:812FDDC2-9BBB-4FFB-8C19-E778CC7ABF16

### Molecular and phylogenetic analysis

One partial ssrDNA sequences of *M. iheringichthys* n. sp. (2001 bp), and 1 partial ssrDNA sequences of *H. avareensis* n. sp. (1933 bp) were obtained. The sequences were obtained from the same plasmodia used for the morphometric analysis. When aligned with each other, the partial sequences of *M. iheringichthys* n. sp. and *H. avareensis* n. sp. showed only 73.3% similarity and differed in 529 nucleotides.

The species genetically most similar to *M. iheringichthys* n. sp. was *Myxobolus cordeiroi* (Adriano et al., 2009), with 95.6% similarity. In turn, the species genetically most similar to *H. avareensis* n. sp. was *Henneguya maculosus* (Carriero et al., 2013), with 93.0% similarity.

**Figure 7.**
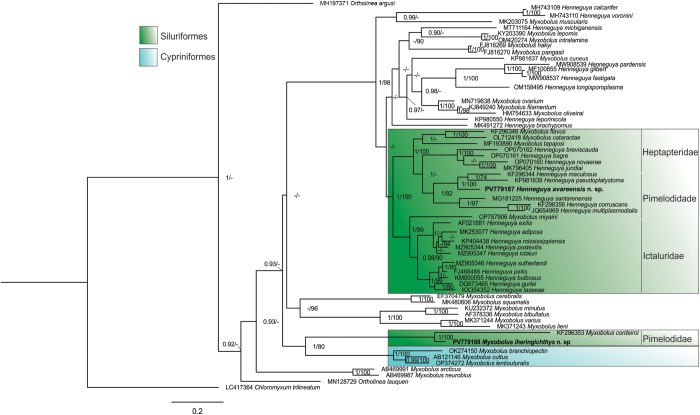
Bayesian inference phylogenetic tree showing the placement of *Henneguya avareensis* n. sp. and *Myxobolus iheringichthys* n. sp. In relation to the genetically most similar species of *Henneguya*/*Myxobolus* obtained from GenBank. The scale bar is given below the tree. The numbers on the branches indicate posterior probability/bootstrap values. Values below 0.9/70 were suppressed with -.

Our phylogenetic analysis retrieved the new myxozoan species in study and their genetically most similar relatives – represented by *Myxobolus and Henneguya* spp. – divided into several subclades (Figure 7). *Myxobolus iheringichthys* n. sp. clusters together with *M. cordeiroi –* another myxobolid that infects a Pimelodidae host – together forming a subclade sister to *Myxobolus* spp. that parasitize freshwater Cypriniformes. In turn, *H. avareensis* n. sp. clusters with *H. maculosus* and *Henneguya pseudoplatystoma* (Naldoni et al., 2009) within a subclade of *Henneguya* spp. that also parasitize the gills of freshwater fish belonging to the family Pimelodidae.

### Remarks

When morphologically compared to *M. cordeiroi, M. iheringichthys* n. sp. showed differences in almost all analysed characteristics, mainly in the width of the myxospore body (7.1–7.5 μm vs 5.5–6.4 μm) and in the length of the polar capsule (5.2–5.4 μm vs 3.7–4.6 μm). Highest morphological similarity was found with *Myxobolus figueirae* (Naldoni et al., 2018) and *Myxobolus sciades* Azevedo, Casal, Mendonça, Carvalho, Mato and Matos, 2010 (Table 1). However, the myxospores of *M. sciades* are narrower (4.3 ± 0.2 μm vs 5.8 ± 0.3 μm) and less thick (2.6 ± 0.3 μm vs 4.4 ± 0.2 μm) than those of *M. iheringichthys n. sp.*, while the myxospores of *M. figueirae* differ in shape, being ovoid instead of round. Additionally, these species differ in host species, with *M. figueirae* further displaying tropism towards the skin.

**Table 1.** Morphometric comparison between *Myxobolus iheringichthys* n. sp. and other *Myxobolus* spp. described from South American siluriformes

Species	BL	BW	T	LP	WP	PTc	Site of infection	Host	Reference
*Myxobolus iheringichthys* n. sp.	8.9 ± 0.3 (8.2–9.5)	5.8 ± 0.3 (5.5–6.4)	4.4 ± 0.2 (4.1–4.8)	4.3 ± 0.3 (3.7–4.6)	2.0 ± 0.2 (1.6–2.4)	6−7	Gills	*Iheringichthys labrosus* (Lütken, 1874)	This study
*Myxobolus niger*	11.3 ± 0.4	6.8 ± 0.2	4.1 ± 0.2	5.0 ± 0.3	2.0 ± 0.1	6−7	Gills	*Corydoras melini* Lönnberg and Rendahl, 1930	Mathews et al. (2016)
*Myxobolus marajoensis*	10.9 (10.0–11.6)	5.1 (4.2–5.4)	–	5.3 ± 0.6	1.6 ± 0.36	–	Intestine	*Rhamdia quelen* (Quoy and Gaimard, 1824)	Abrunhosa et al. (2017)
*Myxobolus figueirae*	9.5 ± 0.3 (9.1–10)	6.4 ± 0.3 (5.8–6.9)	4.5 ± 0.1 (4.4–4.5)	4.1 ± 0.3^a^ 3.2 ± 0.3^b^	2.1 ± 0.3^a^ 1.4 ± 0.2^b^	7−8	Skin	*Phractocephalus hemioliopterus* (Bloch and Schneider, 1801)	Naldoni et al. (2018)
*Myxobolus adrianoi*	22.4 ± 0.3	16.3 ± 0.1	–	14.3 ± 0.2	6.5 ± 0.1	–	Intestine	*Corydoras schwartzi* Rössel, 1963	Mathews et al. (2020)
*Myxobolus cataractae*	7.8 ± 0.4 (7.1–8.9)	5.9 ± 0.4 (5.1–6.6)	3.9 ± 0.3 (3.4–4.4)	3.5 ± 0.2 (3.0–3.9)	1.7 ± 0.2 (1.3–2.1)	6−7	Gills	*Imparfinis mirini* Haseman, 1911	Vieira et al. (2022a)
*Myxobolus imparfinis*	7.9 ± 0.3	5.5 ± 0.5	3.7 ± 0.3	3.9 ± 0.3	1.7 ± 0.1	6−7	Gills	*Imparfinis mirini* Haseman, 1911	Vieira et al. (2018)
*Myxobolus flavus*	9.2 ± 0.2	6.5 ± 0.3	4.2 ± 0.2	4.5 ± 0.2	1.6 ± 0.1	4−5	Gills	*Pseudoplatystoma corruscans* (Spix and Agassiz, 1829)	Carriero et al. (2013)
*Myxobolus tapajosi*	15.0	10.7	–	5.8	3.0	6−7	Gills	*Brachyplatystoma rousseauxii* (Castelnau, 1855)	Zatti et al. (2018)
*Myxobolus sciades*	9.1 ± 0.3	4.3 ± 0.2	2.6 ± 0.3	4.4 ± 0.4	1.4 ± 0.4	9−10	Gills	*Sciades herzbergii* (Bloch, 1794)	Azevedo et al. (2010)

BL: body length; BW: body width; T: thickness; LP: length of polar capsules; WP: width of polar capsules; PTc: number of coils of polar tubules. Measurements are given in μm.

a Largest capsule.

b Smaller capsule.

When morphologically compared to *H. maculosus, H. avareensis* n. sp. showed differences mainly in the length of the caudal appendages (17.5 ± 1.0 μm vs 23.0 ± 1.7 μm) and in the length of the myxospore body (13.7 ± 0.6 μm vs 11.9 ± 0.4 μm). Two other species, *H. pseudoplatystoma* and *Henneguya quelen* (Abrunhosa et al., 2018), presented highest morphological similarity to *H. avareensis* n. sp. (Table 2). However, the myxospore body of *H. quelen* is longer (15.6 ± 0.8 μm vs 11.9 ± 0.4 μm) than that of *H. avareensis* n. sp., while the myxospores of *H. pseudoplatystoma* are narrower (3.4 ± 0.4 μm vs 5.4 ± 0.5 μm) and display shorter polar capsules (3.3 ± 0.4 μm vs 6.4 ± 0.3 μm).

**Table 2.** Morphometric comparison between *Henneguya avareensis* n. sp. and other *Henneguya* spp. described from South American siluriformes

Species	TL	BL	BW	T	CA	LP	WP	PTc	Site of infection	Host	Reference
*Henneguya avareensis* n. sp.	34.8 ± 1.5 (32.3–36.3)	11.9 ± 0.4 (11.2–12.4)	5.4 ± 0.5 (4.7–6.0)	4.1 ± 0.1 (3.9–4.2)	23.0 ± 1.7 (20.0–25.0)	6.4 ± 0.3 (6.0–6.6)	2.1 ± 0.1 (2.0–2.2)	10–12	Gills	*Iheringichthys labrosus* (Lütken, 1874)	This study
*Henneguya santarenensis*	31.9 ± 3 (26.3–36.1)	10.8 ± 0.5 (9.6–11.9)	4.3 ± 0.3 (3.7–4.9)	3.6 ± 0.2 (3.4–3.7)	21.0 ± 3.1 (16.6–25.6)	4.6 ± 0.4 (3.8–5.5)	1.4 ± 0.2 (1.0–1.7)	15	Gills	*Phractocephalus hemioliopterus* (Bloch and Schneider, 1801)	Naldoni et al. (2018)
*Henneguya novaerae*	24.9 ± 0.9 (23.3–26.8)	10.7 ± 0.5 (10.1–11.6)	3.8 ± 0.3 (3.2–4.2)	3.5 ± 0.3 (3.1–4.0)	14.7 ± 1.4 (11.5–16.1)	4.8 ± 0.4 (3.8– 5.5)	1.3 ± 0.2 (1.1–1.7)	6	Gills	*Rhamdia quelen* (Quoy and Gaimard, 1824)	Vieira et al. (2022b)
*Henneguya bagre*	20.9 ± 0.9 (19.2–21.8)	11.2 ± 0.5 (10.5–12.4)	5.1 ± 0.3 (4.6 –6.1)	3.8 ± 0.2 (3.6–4.1)	9.8 ± 0.9 (8.2–11.1)	5.9 ± 0.6 (4.9–6.7)	1.6 ± 0.1 (1.4–1.8)	7	Gills	*Rhamdia quelen* (Quoy and Gaimard, 1824)	Vieira et al. (2022b)
*Henneguya breviscauda*	17.1 ± 0.9 (15.9–18.7)	10.7 ± 0.4 (9.6 – 10.8)	5.3 ± 0.5 (4.4–6.1)	3.7 ± 0.1 (3.6–3.9)	7.2 ± 0.8 (5.8 – 8.2)	5.4 ± 0.2 (5.1 – 5.6)^a^ 4.6 ± 0.3 (4.2–5.0)^b^	1.5 ± 0.1 (1.2–1.6)^a^ 1.2 ± 0.1 (1.1 – 1.3)^b^	7	Gills	*Rhamdia quelen* (Quoy and Gaimard, 1824)	Vieira et al. (2022b)
*Henneguya jundiai*	26.9 ± 1.9 (22.9–29.2)	9.5 ± 0.4 (8.8 – 10.0)	4.6 ± 0.4 (4.1–5.5)	–	17.3 ± 1.8 (14.1–19.8)	4.9 ± 0.3 (4.6–5.5)	1.4 ± 0.2 (1.2–1.7)	6–7	Gills	*Rhamdia quelen* (Quoy and Gaimard, 1824)	Negrelli et al. (2019)
*Henneguya quelen*	40.0 ± 2.8 (37.0–42.8)	15.6 ± 0.8 (14.3–16.4)	4.1 ± 0.3 (3.9–4.4)	–	24.3 ± 2.2 (21 – 26.5)	5.5 ± 0.5 (5.2−6.0)	1.6 ± 0.2 (1.4–1.8)	–	Kidney	*Rhamdia quelen* (Quoy and Gaimard, 1824)	Abrunhosa et al. (2018)
*Henneguya rhamdia*	50.0 ± 1.8	13.1 ± 1.1	5.2 ± 0.5	2.5 ± 0.5	36.9 ± 1.6	4.7 ± 0.4	1.1 ± 0.2	10–11	Gills	*Rhamdia quelen* (Quoy and Gaimard, 1824)	Matos et al. (2005)
*Henneguya guanduensis*	27.3 ± 38.1	11.4 ± 16.7	4.9 ± 7.9	–	15.6 ± 22.5	4.4 (3.3–5.6)^a^ 4.1 (3.3–5.3)^b^	2.0 (1.6–2.3)^a^ 2.2 (1.5–2.8)^b^	3–6	Gills	*Hoplosternum littorale* (Hancock, 1828)	Abdallah et al. (2007)
*Henneguya pseudoplatystoma*	33.2 ± 1.9	10.4 ± 0.6	3.4 ± 0.4	–	22.7 ± 1.7	3.3 ± 0.4	1.0 ± 0.1	6–7	Gills	*Pseudoplatystoma corruscans* (Spix and Agassiz, 1829) and *Pseudoplatystoma fasciatum* (Linnaeus, 1766)	Naldoni et al. (2009)
*Henneguya eirasi*	37.1 ± 1.8	12.9 ± 0.8	3.4 ± 0.3	3.1 ± 0.1	24.6 ± 2.2	5.4 ± 0.5	0.7 ± 0.1	12–13	Gills	*Pseudoplatystoma corruscans* (Spix and Agassiz, 1829) and *Pseudoplatystoma fasciatum fasciatum* (Linnaeus, 1766)	Naldoni et al. (2011)
*Henneguya multiplasmodialis*	30.8 ± 1.3	14.7 ± 0.5	5.2 ± 0.3	4.4 ± 0.1	15.4 ± 1.3	6.1 ± 0.1	1.4 ± 0.1	6–7	Gills	*Pseudoplatystoma corruscans* (Spix and Agassiz, 1829)	Adriano et al. (2012)
*Henneguya corruscans*	27.6 (25–29)	14.3 (13–15)	5.0	3.1 ± 0.4	13.7 (12–15)	6.8 (6–7)	2.0	5–6	Gills	*Pseudoplatystoma corruscans* (Spix and Agassiz, 1829)	Eiras et al. (2009)

TL: total length; BL: body length; BW: body width; T: thickness; CA: length of caudal appendages; LP: length of polar capsules; WP: width of polar capsules; PTc: number of coils of polar tubules. Measurements are given in μm.

a Largest capsule.

b Smaller capsule.

None of the other myxobolid species used for comparison exhibited morphological or morphometric characteristics resembling the new species being described here. They differed in at least 1 morphometric aspect, such as the number of turns of the polar tubules (varying by at least 3 turns), or in their partial genetic sequences.

## Discussion

For many years, the taxonomic classification of myxozoans was based solely on morphological and morphometric characteristics of the plasmodia and myxospores. Since the 1990s, molecular tools have been implemented, aiding the correct identification of myxozoans species, with a great number of species described since then (Negrelli et al., 2019; Úngari et al., 2019; Vieira et al., 2020). Increasing amounts of sequence data have confirmed the paraphyletic nature of the genus *Myxobolus*, with several *Henneguya* species clustering within the *Myxobolus* clade (Xiao and Desser, 2000; Fiala, 2006; Liu et al., 2010; Vieira et al., 2019), as observed in this study. Eszterbauer (2004) found that a preference for a specific developmental site, rather than spore morphology, is a significant criterion for determining phylogenetic relationships between *Myxobolus* species. The phylogenetic analysis performed in this study agrees with previous cladograms, through supporting host (order or family level) and tissue tropism as robust phylogenetic signals for clustering Myxobolidae species (Carriero et al., 2013; Capodifoglio et al., 2016; Vieira et al., 2019, 2021). *Henneguya avareensis* n. sp. clusters within a subclade that is exclusively composed by species that parasitize siluriforms of the family Pimelodidae, being an integral part of a large clade formed by myxozoan parasites of Siluriformes. In turn, *M. iheringichthys* n. sp. is included in a subclade formed by *M. cordeiroi* – a species that parasitizes multiple organs of *Zungaro jahu* (Ihering, 1898) (Siluriformes: Pimelodidae) – and myxobolids that parasitize Cypriniformes. *Myxobolus cordeiroi* was the first species of *Myxobolus* described in Brazil using molecular and phylogenetic analysis (Adriano et al., 2009). The phylogenetic analysis of the species description already includes species that parasitize Cypriniformes, which shows a possible co-relationship between these orders of hosts and their myxozoan parasites (Adriano et al., 2009). *Myxobolus cordeiroi* appears in an isolated subclade and may reflect the existence of a lineage of myxozoans parasites of fish from South America or a lineage of *Myxobolus* parasites of Pimelodidae. In this study, *M. iheringichthys* n. sp. appears as a sister species of *M. cordeiroi*.

The specific localization of plasmodia in the hosts tissues is a significant taxonomic trait for distinguishing among histozoic myxozoan species (Molnár, 2002). Regrettably, due to the limited number of infected hosts analysed in this study, molecular identifications took precedence over histological preparations. Subsequent research should focus on identifying the specific tissue within the gills serving as the site of infection for the new species.

In the current study, 2 new species of myxozoans were described based on morphometric characteristics, supported by molecular and phylogenetic analyses. The evidence collected conclusively confirmed the existence of 2 new species, identified as *Henneguya avareensis* n. sp. and *Myxobolus iheringichthys* n. sp., infecting the gills of *I. labrosus*. Thus, we advocate for ongoing surveillance of these parasites in farmed stocks of *I. labrosus* to assess potential pathogenic effects they might induce.
